# Supplementary material for: The implications of parent mental health and wellbeing for parent-child attachment: A systematic review
Source: PLoS One. 2021 Dec 16;16(12):e0260891. doi: 10.1371/journal.pone.0260891 (PMC8675743; doi:10.1371/journal.pone.0260891)
Supplement: S1 File — (PDF) [file pone.0260891.s002.pdf]

NHS
National Institute for Health Research

UNIVERSITY of York
Centre for Reviews and Dissemination

Systematic review

1. * Review title.

Give the working title of the review, for example the one used for obtaining funding. Ideally the title should state succinctly the interventions or exposures being reviewed and the associated health or social problems. Where appropriate, the title should use the PI(E)COS structure to contain information on the Participants, Intervention (or Exposure) and Comparison groups, the Outcomes to be measured and Study designs to be included.

The impact of parent mental health and trauma on the transmission of adult attachment to parent-infant attachment

2. Original language title.

For reviews in languages other than English, this field should be used to enter the title in the language of the review. This will be displayed together with the English language title.

English

3. * Anticipated or actual start date.

Give the date when the systematic review commenced, or is expected to commence.

01/01/2020

4. * Anticipated completion date.

Give the date by which the review is expected to be completed.

31/12/2020

5. * Stage of review at time of this submission.

Indicate the stage of progress of the review by ticking the relevant Started and Completed boxes. Additional information may be added in the free text box provided.
Please note: Reviews that have progressed beyond the point of completing data extraction at the time of initial registration are not eligible for inclusion in PROSPERO. Should evidence of incorrect status and/or completion date being supplied at the time of submission come to light, the content of the PROSPERO record will be removed leaving only the title and named contact details and a statement that inaccuracies in the stage of the review date had been identified.
This field should be updated when any amendments are made to a published record and on completion and publication of the review. If this field was pre-populated from the initial screening questions then you are not able to edit it until the record is published.

The review has not yet started: Yes

Review stage	Started	Completed
Preliminary searches	No	No
Piloting of the study selection process	No	No
Formal screening of search results against eligibility criteria	No	No
Data extraction	No	No
Risk of bias (quality) assessment	No	No
Data analysis	No	No

Provide any other relevant information about the stage of the review here (e.g. Funded proposal, protocol not yet finalised).

6. * Named contact.

The named contact acts as the guarantor for the accuracy of the information presented in the register record.

Alixandra Risi

Email salutation (e.g. "Dr Smith" or "Joanne") for correspondence:

Miss Risi

7. * Named contact email.

Give the electronic mail address of the named contact.

anr652@uowmail.edu.au

8. Named contact address

Give the full postal address for the named contact.

Northfields Ave, Wollongong NSW 2522

9. Named contact phone number.

Give the telephone number for the named contact, including international dialling code.

10. * Organisational affiliation of the review.

Full title of the organisational affiliations for this review and website address if available. This field may be completed as 'None' if the review is not affiliated to any organisation.

University of Wollongong

Organisation web address:

https://www.uow.edu.au

11. * Review team members and their organisational affiliations.

Give the title, first name, last name and the organisational affiliations of each member of the review team.

Affiliation refers to groups or organisations to which review team members belong.

Miss Alixandra Risi. University of Wollongong
Dr Amy Bird. University of Wollongong, University of Waikato
Dr Judy Pickard. University of Wollongong
Assistant/Associate Professor Jane Herbert. University of Wollongong

12. * Funding sources/sponsors.

Give details of the individuals, organizations, groups or other legal entities who take responsibility for initiating, managing, sponsoring and/or financing the review. Include any unique identification numbers assigned to the review by the individuals or bodies listed.

University of Wollongong

13. * Conflicts of interest.

List any conditions that could lead to actual or perceived undue influence on judgements concerning the main topic investigated in the review.

None

14. Collaborators.

Give the name and affiliation of any individuals or organisations who are working on the review but who are not listed as review team members.

15. * Review question.

State the question(s) to be addressed by the review, clearly and precisely. Review questions may be specific or broad. It may be appropriate to break very broad questions down into a series of related more specific questions. Questions may be framed or refined using PI(E)COS where relevant.

Does parent mental health and trauma impact the transmission of adult attachment to parent-infant attachment?

16. * Searches.

State the sources that will be searched. Give the search dates, and any restrictions (e.g. language or publication period). Do NOT enter the full search strategy (it may be provided as a link or attachment.)

Searches will be conducted across 6 academic databases, including: PsycINFO, PsycARTICLES, MEDLINE, Scopus, SocINDEX, and ERIC.

No restriction will be placed on the year of publication. However, all articles that are irrelevant to the research question, have no been peer-review, or are written in a language other than English will be excluded.

Keywords will be developed for each element of the research question. The keyword will include commonly used terminology, as well as alternative terms and synonyms. In addition to database searching, the reference lists of relevant review articles will also be scanned for potential applicable studies.

17. URL to search strategy.

Give a link to a published pdf/word document detailing either the search strategy or an example of a search strategy for a specific database if available (including the keywords that will be used in the search

strategies), or upload your search strategy.Do NOT provide links to your search results.

https://www.crd.york.ac.uk/PROSPEROFILES/157247_STRATEGY_20191209.pdf

Alternatively, upload your search strategy to CRD in pdf format. Please note that by doing so you are consenting to the file being made publicly accessible.

Do not make this file publicly available until the review is complete

18. * Condition or domain being studied.

Give a short description of the disease, condition or healthcare domain being studied. This could include health and wellbeing outcomes.

This review will examine attachment transmission, and whether parental mental health and trauma impacts the transmission of adult attachment to parent-infant attachment. Mental health is defined as an individual's psychological and emotional well-being and includes factors such as depression, anxiety, stress, personality disorders, mindfulness, reflective functioning, and mentalising. Trauma refers to distressing experiences. Terms used to encompass trauma include PTSD, complex PTSD, chronic trauma, and adverse childhood experiences (ACE).

19. * Participants/population.

Give summary criteria for the participants or populations being studied by the review. The preferred format includes details of both inclusion and exclusion criteria.

Included: Parents and children (aged from infancy to adolescence).

Excluded: Parents and children older than adolescence.

20. * Intervention(s), exposure(s).

Give full and clear descriptions or definitions of the nature of the interventions or the exposures to be reviewed.

As described in Q18.

21. * Comparator(s)/control.

Where relevant, give details of the alternatives against which the main subject/topic of the review will be compared (e.g. another intervention or a non-exposed control group). The preferred format includes details of both inclusion and exclusion criteria.

There will be no comparator or control used in this review.

22. * Types of study to be included.

Give details of the types of study (study designs) eligible for inclusion in the review. If there are no restrictions on the types of study design eligible for inclusion, or certain study types are excluded, this should be stated. The preferred format includes details of both inclusion and exclusion criteria.

Both quantitative and qualitative studies will be included in this review, and there will be no restriction placed on the year of publication. However, all articles that are irrelevant to the research question, have not been peer-review, or are written in a language other than English will be excluded. Review articles will be excluded, although the reference lists of relevant review articles will be scanned to check for additional studies, as outlined in the search strategy.

23. Context.

Give summary details of the setting and other relevant characteristics which help define the inclusion or exclusion criteria.

24. * Main outcome(s).

Give the pre-specified main (most important) outcomes of the review, including details of how the outcome is defined and measured and when these measurement are made, if these are part of the review inclusion criteria.

The main outcome of this review is intergenerational transmission of attachment. This may include transmission of attachment classification - secure / insecure - as well as attachment security. From a parent perspective this may be measured using the Adult Attachment Interview (AAI), Experiences in Close Relationships (ECR), or Relationships Questionnaire (RQ) or similar. From an infant / child / adolescent perspective this may include the Strange Situation Protocol (SSP), the Attachment Q-Set; and/or a validated and reliable narrative story stem task.

Timing and effect measures

-

25. * Additional outcome(s).

List the pre-specified additional outcomes of the review, with a similar level of detail to that required for main outcomes. Where there are no additional outcomes please state 'None' or 'Not applicable' as appropriate to the review

Not applicable

Timing and effect measures

Not applicable

26. * Data extraction (selection and coding).

Describe how studies will be selected for inclusion. State what data will be extracted or obtained. State how this will be done and recorded.

One researcher will independently screen all articles arising from the search via their titles and abstracts identifying whether or not the papers in the sample are likely to be relevant to the research question.

Following this process, full-text articles will be retrieved for all records deemed to meet the eligibility criteria (and for all records where further information is required in order to make an appropriate judgement).

Covidence will be used for this assessment process. These full text articles will be independently assessed for eligibility by two separate researchers, based on the specified inclusion and exclusion criteria. Inter-rater reliability for the inclusion/exclusion of full-text articles will be assessed and reported in the final write-up of the study, and all inconsistencies in the included/excluded articles will be discussed with a third reviewer.

The third reviewer will make the final decision on each discrepant article.

Data extracted from eligible full-text articles will include: participant numbers and demographics; mental

health status; trauma experiences; measure/s of attachment; and attachment transmission.

27. * Risk of bias (quality) assessment.

Describe the method of assessing risk of bias or quality assessment. State which characteristics of the studies will be assessed and any formal risk of bias tools that will be used.

As two separate researchers will independently screen all full-text articles for inclusion. the likelihood of bias in the selection or articles will be reduced. Additionally, the quality of all included articles will be valued by two researchers via the Appraisal Tool for Cross-Sectional Studies (AXIS). Through this process, any studies which appear to contain methodological flows or potentially compromised results will be noted and further discussed in the synthesisation of findings.

28. * Strategy for data synthesis.

Provide details of the planned synthesis including a rationale for the methods selected. This **must not be generic text** but should be **specific to your review** and describe how the proposed analysis will be applied to your data.

Given the likely variability in study design, measures, and participants, the aim of the current review is to provide a thematic analysis fo all relevant literature in order to understand how mental health and trauma influence the transmission of adult attachment to parent-infant attachment. This synthesis will highlight any clear commonalities and categorise the reported influences.

29. * Analysis of subgroups or subsets.

State any planned investigation of ‘subgroups’. Be clear and specific about which type of study or participant will be included in each group or covariate investigated. State the planned analytic approach.

No analysis of subgroups is being undertaken.

30. * Type and method of review.

Select the type of review and the review method from the lists below. Select the health area(s) of interest for your review.

Type of review

Cost effectiveness
No

Diagnostic
No

Epidemiologic
No

Individual patient data (IPD) meta-analysis
No

Intervention
No

Meta-analysis
No

Methodology
No

Narrative synthesis

No

Network meta-analysis
No

Pre-clinical
No

Prevention
No

Prognostic
No

Prospective meta-analysis (PMA)
No

Review of reviews
No

Service delivery
No

Synthesis of qualitative studies
No

Systematic review
Yes

Other
No

Health area of the review

Alcohol/substance misuse/abuse
No

Blood and immune system
No

Cancer
No

Cardiovascular
No

Care of the elderly
No

Child health
No

Complementary therapies
No

Crime and justice
No

Dental
No

Digestive system
No

Ear, nose and throat
No

Education
No

Endocrine and metabolic disorders
No

Eye disorders

No

General interest
No

Genetics
No

Health inequalities/health equity
No

Infections and infestations
No

International development
No

Mental health and behavioural conditions
Yes

Musculoskeletal
No

Neurological
No

Nursing
No

Obstetrics and gynaecology
No

Oral health
No

Palliative care
No

Perioperative care
No

Physiotherapy
No

Pregnancy and childbirth
No

Public health (including social determinants of health)
No

Rehabilitation
No

Respiratory disorders
No

Service delivery
No

Skin disorders
No

Social care
No

Surgery
No

Tropical Medicine
No

Urological
No

Wounds, injuries and accidents
No

Violence and abuse
No

31. Language.

Select each language individually to add it to the list below, use the bin icon to remove any added in error.
English

There is not an English language summary

32. * Country.

Select the country in which the review is being carried out from the drop down list. For multi-national collaborations select all the countries involved.

Australia

33. Other registration details.

Give the name of any organisation where the systematic review title or protocol is registered (such as with The Campbell Collaboration, or The Joanna Briggs Institute) together with any unique identification number assigned. (N.B. Registration details for Cochrane protocols will be automatically entered). If extracted data will be stored and made available through a repository such as the Systematic Review Data Repository (SRDR), details and a link should be included here. If none, leave blank.

34. Reference and/or URL for published protocol.

Give the citation and link for the published protocol, if there is one

Give the link to the published protocol.

Alternatively, upload your published protocol to CRD in pdf format. Please note that by doing so you are consenting to the file being made publicly accessible.

No I do not make this file publicly available until the review is complete

Please note that the information required in the PROSPERO registration form must be completed in full even if access to a protocol is given.

35. Dissemination plans.

Give brief details of plans for communicating essential messages from the review to the appropriate audiences.

Do you intend to publish the review on completion?

Yes

36. Keywords.

Give words or phrases that best describe the review. Separate keywords with a semicolon or new line. Keywords will help users find the review in the Register (the words do not appear in the public record but are included in searches). Be as specific and precise as possible. Avoid acronyms and abbreviations unless these are in wide use.

37. Details of any existing review of the same topic by the same authors.

Give details of earlier versions of the systematic review if an update of an existing review is being registered, including full bibliographic reference if possible.

38. * Current review status.

Review status should be updated when the review is completed and when it is published. For newregistrations the review must be Ongoing.
Please provide anticipated publication date

Review_Ongoing

39. Any additional information.

Provide any other information the review team feel is relevant to the registration of the review.

40. Details of final report/publication(s).

This field should be left empty until details of the completed review are available.

Give the link to the published review.
