# Supplementary material for: The implications of parent mental health and wellbeing for parent-child attachment: A systematic review
Source: PLoS One. 2021 Dec 16;16(12):e0260891. doi: 10.1371/journal.pone.0260891 (PMC8675743; doi:10.1371/journal.pone.0260891)
Supplement: Attachment — Submitted filename: Response to Reviewers.docx [file pone.0260891.s003.docx]

25^th^ October 2021

Dr Soo Hyun Rhee

Academic Editor

*PLOS ONE*

Dear Dr Rhee,

Thank you for taking the time to review our manuscript ‘The impact of parent mental health on the intergenerational transmission of attachment: A systematic review’ and inviting us to submit a revised version. We are extremely grateful to have had two attachment experts review and provide feedback on our manuscript. We appreciate that each reviewer held different interpretations of our paper. Although different views were held by each reviewer, their comments highlighted areas in our manuscript that required revisions and assisted us in developing an improved manuscript. We believe that these revisions better acknowledge the limits of this systematic review, and we hope that this research will be – as noted by Reviewer 2 - of interest to many attachment researchers.

Please see below our responses to the comments provided by the reviewers.

**Reviewer 1**

*Point 1: It is not obvious that psychopathology is the place to look for additional predictive power across generations here. If it turned out that psychopathology contributed to predicting attachment across generations it would not really be a specific contribution to attachment theory. It would just be a generic effect that the disorganization associated with psychopathology has on so many other variables. For example, psychopathology might account in part for inconsistency in arithmetic scores from year to year. What attachment theorists are looking for would be something specifically related to attachment processes, e.g., contribution from the other parent, marital variables, additional demands on the mother such as employment, etc. Something that goes to the processes the theory implicates in attachment development. Even if psychopathology were statistically a significant factor (see below) it would simply raise the question what aspects of caregiving mediate the effect. At that point we would be right back where we are because the problem in not being able to predict attachment security across-generations seems to lie in not knowing what aspects of care (other than sensitivity) are causal. In brief, even if the study had found significant effects related to psychopathology, this would be of limited interest to attachment theorists.*

The first point raised by Reviewer 1 questions whether examining psychopathology as a mediator for attachment transmission is useful and suggests that if psychopathology did contribute to predicting attachment across generations, it would not contribute significantly to attachment theory. We apologise for our lack of clarity which lead to confusion about the scope of this study. To clarify, through our systematic review, we were investigating the current literature surrounding the implications of parent mental health and wellbeing on parent-child attachment. We can understand how especially Figure 1 and the explanations related to that could read as an attempt to reconceptualise attachment theory. We still believe that it is important to understand the role of mental health and wellbeing in attachment transmission. While much observational research has been conducted with community samples, we must also understand how attachment transmission presents in non-community samples as this is where much of attachment-informed intervention work occurs. In addition, the interventions for the treatment of parental mental health as opposed to dyadic interaction are quite distinct. Therefore, if psychopathology and positive mental health constructs were found to play any role in attachment transmission, this could have significant clinical implications. We recognise some of our wording used throughout the manuscript was unclear about the scope of our study. We have now modified the title of our manuscript to de-emphasise the theoretical component of the study. The revised title reads ‘The implications of parent mental health and wellbeing on parent-child attachment: A systematic review’. To provide increased clarity about the role mental health and wellbeing may play in the relationship between parent and infant attachment, we have removed Figure 1 and updated Figure 2 (now Figure 1). This figure better proposes that the potential mediating or moderating role of mental health or wellbeing is transmitted through differences in caregiving behavior. Finally, we have reduced the emphasis of our systematic review ‘closing the transmission gap’ by modifying our language throughout the manuscript (e.g., see p.2, p.11, pp.38-39).

Additionally, Reviewer 1 suggests that if psychopathology did contribute to predicting attachment transmission, it would be due to the relationship between psychopathology and attachment disorganisation. We agree that psychopathology and attachment disorganisation are related. However, we also think that viewing psychopathology and attachment disorganization as distinct constructs may have some value in the following ways:

- Psychopathology and disorganisation are related differentially to child outcomes. The distinction between psychopathology and disorganisation is demonstrated by longitudinal research showing psychopathology is a correlate of continuity and discontinuity in attachment. Results stemming from the Minnesota Longitudinal Study of Parents and Children demonstrate disruptions in attachment across time can be associated with parental psychopathology (e.g., Sroufe, 2005; Sroufe, Coffino, & Carlson, 2010; Sroufe, Eyeland, & Kreutzer, 1990; Weinfield, Sroufe, & Egeland, 2000). Changes in parental stress and/or depression were found to relate to continuity and change in attachment classification from infancy to adulthood in a high-risk sample. Individuals with secure early attachment histories may show a rebound to better functioning after setbacks (e.g., parental stress/depression decreases). These findings highlight the distinction between psychopathology and attachment classification and provide a strong rationale for examining parental psychopathology as a mediator in attachment transmission.
- It is possible to have episodes of psychopathology without necessarily leading to attachment disorganisation, and vice versa. For example, an individual may experience episodes of depression or may have a genetic predisposition to schizophrenia, however these do not automatically, in themselves, infer attachment disorganisation. For example, Riggs et al. (2007) found both proportions of secure and insecure AAI classifications were represented in a psychiatric sample of trauma survivors.

We would also like to highlight that our systematic review does not focus solely on examining disorganised versus organised attachment and considers all attachment classifications. It also extends beyond psychopathology and considers mental wellbeing to examine the full continuum of mental health. Examining parent wellbeing constructs allows us to consider how the internal resources of the parent (e.g., mindfulness, reflective functioning) relate to attachment processes. Reviewer 1 comments that even if psychopathology was a statistically significant factor, it would simply raise the question what aspects of caregiving mediate the effect. As demonstrated in the results and discussion of our systematic review, reflective functioning and other positive mental health constructs may mediate this effect. Mental health lies along a continuum with positive wellbeing and the consideration of these variables may provide answers to some of the queries raised by Reviewer 1. Upon reflection, we acknowledge we did not emphasize the inclusion of mental wellbeing enough in our initial manuscript. We have modified our manuscript to ensure parent mental health and wellbeing are equally emphasised throughout. For example, instead of using only ‘parent mental health’ when talking about the constructs examined throughout our systematic review, we now use the phrase ‘parent mental health and wellbeing’. To ensure it is clear to readers that we are examining the complete continuum of mental health in our systematic review, we have also modified the title of our manuscript to include ‘wellbeing’.

*Point 2: The authors find only a few studies from which to extract data. This probably reflects the point mentioned above. Moreover, the few studies use a variety of different attachment measures. The adult measures, in particular, as absolutely not comparable. Despite being entitles "attachment measures" by the people who developed them, there is no evidence that they measure the same constructs. Indeed, there is considerable evidence that they are barely if at all inter-correlated and even this small association is easily accounted for by nuisance variables. In brief, having searched for studies on which to conduct their study, the authors could have determined without any statistical analysis at all that the literature does not support any useful conclusions regarding psychopathology as a mediator of cross-generation attachment transmission.*

We are in full agreement with Reviewer 1 that the variety of attachment measures used across the studies included in the systematic review do not measure the same constructs. We recognise that although a variety of measures may be described as attachment measures, evidence suggests they are not actually measuring the same construct. The literature, however, has not always clearly made this distinction. As this is a systematic review and not a meta-analysis, it is important that all studies which report they are measuring ‘attachment’ be included in the review. In the beginning stages of our systematic review, we did consider only examining literature that used gold-standard measures of attachment. However, we thought it would be helpful for readers to understand that this body of literature utilises gold-standard measures as well as indirect measures that may reflect a different construct. The inclusion of all studies in our systematic review allowed us draw conclusions about the state of the literature being limited due to the variety of measures which capture different constructs. To provide clarity for future readers, we have made several revisions in our discussion on the differences in attachment measures. Specifically, we have extended our discussion regarding measurement differences in the Introduction (see pp.5-6), provided further justification for including all measures in the Method (see p.13), and elaborated on the importance of understanding how attachment has been measured in the literature in the Discussion (see p.33).

*Point 3: Attachment theorists have gone to some lengths to figure out why cross-generation transmission of attachment security is not as strong as they expect. Numerous studies have examined the issue. In addition, there are numerous conceptual analyses of the problem. Importantly, almost all of this literature refers to work on populations in which psychopathology occurs at very low levels. While one might find some additional significant cross-generational variance in samples that are more diverse with respect to psychopathology, as mentioned above, this would simply reflect generic (rather than attachment specific) effects. It would have little relevance to the problem attachment researchers have set themselves, which is to understand why there is not more cross-generation transmission in ordinary samples.*

In their final point, Reviewer 1 states attachment researchers are interested in understanding why there is not more cross-generational transmission of attachment in community/low-risk samples, and thus, suggests our systematic review will not make a significant contribution considering psychopathology occurs in low levels in such samples. We recognise that the investigation of attachment transmission across all samples is important. However, we believe the examination of attachment transmission in high-risk samples is also of importance. Indeed, several important attachment findings have come from high-risk samples (e.g., Sroufe, 2005; Weinfield, Sroufe, & Egeland, 2000). As noted above, attachment-based interventions are also focused on high-risk and clinical samples because higher rates of insecure attachment occur in these populations. Understanding the variables that affect attachment can inform the development of interventions that can mitigate attachment issues, emphasizing the importance of our review.

**Reviewer 2**

*Point 1: In table 4 (p.21-24), I believe it is relevant to include information about the statistical analysis performed in each study. Particularly, because the authors discuss this topic.*

We thank reviewer two for this suggestion and have now included information about the statistical analyses performed in each study in Table 4.

*Point 2: In the abstract, the authors claim that is difficult to draw conclusions based on the published articles, I agree. However, I think they should alight the most significant results and directions found in their review, otherwise, the readers lose interest after reading the abstract. For instance, "Thus, anxiety may function as a serial mediator, interfering with a parent’s ability to sensitively respond to their infant resulting in insecure parent-child attachments." (p. 38). As well as results on parental substance abuse, depression or trauma.*

We have made modifications to the results section of the Abstract with Reviewer 2’s suggestions in mind to highlight the most significant results and directions found in the review.

**Editor comments**

*Comment 1: Please ensure that your manuscript meets PLOS ONE's style requirements, including those for file naming.*

We have reviewed our manuscript to ensure it meets PLOS ONE’s style requirements.

*Comment 2: We note that you have referenced (ie. Bewick et al. [5]) which has currently not yet been accepted for publication. Please remove this from your References and amend this to state in the body of your manuscript as detailed online in our guide for authors.*

We have reviewed our manuscript and ensured that any unpublished manuscripts are referenced and cited in accordance with PLOS ONE’s referencing guidelines.

*Comment 3: We note that this manuscript is a systematic review or meta-analysis; our author guidelines therefore require that you use PRISMA guidance to help improve reporting quality of this type of study. Please upload copies of the completed PRISMA checklist as Supporting Information with a file name “PRISMA checklist”.*

Accompanying this Response to Reviewers letter, we have uploaded a copy of the completed PRISMA checklist as Supporting Information.

*Comment 4: PLOS ONE does not copy edit accepted manuscripts (https://journals.plos.org/plosone/s/criteria-for-publication#loc-5). To that effect, please ensure that your submission is free of typos and grammatical errors. *Please note that your quality assessment table (Table 1) appears to be incomplete. During your revision, please include a completed version.*

We apologize for and recognize that our quality assessment table (Table 1) appeared to be incomplete. To rectify this, we have now ensured the ticks are visible to symbolize when the criteria was met. We have also included crosses to demonstrate when the criteria were not met (p. 16).

We again thank the reviewers for their thoughtful feedback, which has been extremely helpful in assisting us to develop an improved manuscript. We believe the changes that have been made have improved the quality and clarity of the manuscript. We hope you find we have satisfactorily addressed the points raised during the review process and thank you again for your consideration for publication in PLOS ONE.

Warmly,

**Alixandra Risi**

School of Psychology

University of Wollongong, Australia

Email address: [anr652@uowmail.edu.au](mailto:anr652@uowmail.edu.au)
